# Energy expenditure for isometric contractions of right and left ventricular trabeculae over a wide range of frequencies at body temperature

**DOI:** 10.1038/s41598-019-45273-1

**Published:** 2019-06-20

**Authors:** Toan Pham, Callum M. Zgierski-Johnston, Kenneth Tran, Andrew J. Taberner, Denis S. Loiselle, June-Chiew Han

**Affiliations:** 1 Auckland Bioengineering Institute, The University of Auckland, Auckland, New Zealand; 2 Department of Physiology, The University of Auckland, Auckland, New Zealand; 3 Institute for Experimental Cardiovascular Medicine, University Heart Center Freiburg-Bad Krozingen and Faculty of Medicine, University of Freiburg, Freiburg, Germany; 4 Department of Engineering Science, The University of Auckland, Auckland, New Zealand

**Keywords:** Cardiovascular biology, Bioenergetics

## Abstract

We studied the energy expenditure of isometric contractions using both right-ventricular (RV) and left-ventricular (LV) trabeculae isolated from the rat heart. The energy expenditure under isometric contraction presents entirely as heat liberation. Preparations were challenged to perform at various rates of energy demand while accounting for their inevitable time-dependent decline of performance. They were electrically stimulated to contract at 37 °C with a frequency order (between 0.1 Hz and 10 Hz) dictated by a fully-balanced Latin-Square experimental design. We measured, simultaneously, their stress production and heat output. As functions of stimulus frequency, active stress and heat were not significantly different between RV and LV trabeculae. However, contraction kinetics, indexed as the maximal rate of rise and fall of twitch, were lower in the LV trabeculae. The ratio of heat to stress was greater in the LV trabeculae, suggesting that the economy of contraction of the LV trabeculae is lower. Their lower economy became more pronounced at high stimulus frequencies. Our results allow us to assess whether slowing of kinetics is a causative mechanism for improvement of economy of contraction.

## Introduction

In the mammalian heart, the left ventricle (LV) powers the systemic circulation while the right ventricle (RV) ejects into the pulmonary circulation. The LV develops about a four-fold higher systolic pressure than that of the RV, consistent with its thicker wall, to overcome the higher systemic vascular resistance. In association with its higher systolic pressure, the energy expenditure in terms of oxygen consumption per mass of the LV is double that of the RV^1,2^.

It is acknowledged that cardiac muscles isolated from both ventricles are capable of developing the same peak isometric systolic stress. This finding obtains, provided that isolated LV and RV preparations are both subjected to the same experimental conditions. Evidence to support this finding has been reported across various species including the rat^3–5^, dog^6^ and human^7^, and over a range of stimulus frequencies. Nevertheless, it is less certain whether the energy expenditure of isolated cardiac preparations is the same between ventricles, specifically when compared across a range of stimulus frequencies. To date, it has only been shown that at a single stimulus frequency, one which resembles the physiological heart rate of the species under investigation, isolated trabeculae from either ventricle of the rat have the same heat output at body temperature^8–10^.

The principal aim of this study was thus to explore, as functions of both ventricle and stimulus frequency, the heat output of rat cardiac muscle preparations. We have chosen to subject isolated ventricular trabeculae to isometric contractions for the basis of comparison, given that the energy expenditure transpires entirely as heat. The heat liberated during isometric contractions arises from hydrolysis of adenosine triphosphate (ATP) by various ATPases (initial heat) and the resynthesis of ATP by the mitochondria (recovery heat). The former are principally the sarcoplasmic reticular Ca^2+^-ATPase (SERCA), the membrane-bound Na^+^-K^+^-ATPase and (indirectly) the Na^+^-Ca^2+^ exchanger, that collectively synchronise the cycling of Ca^2+^ among cellular compartments, thereby affecting the Ca^2+^-triggered, actin-activated, myosin ATPase.

We have designed a set of experiments to allow separate assessment of two effects: stimulus frequency on RV and LV trabeculae, and the unavoidable time-dependent decline of mechanical performance of isolated cardiac muscle preparations. Preparations isolated from the rat heart in particular exhibit a run-down of force development with time, which is more pronounced at body temperature. The initially negative force-frequency relation of isolated rat papillary muscles has been reported to become positive 3–4 hours post-excision at which time the developed force across a range of frequencies has fallen to about one-half of its initial value – a phenomenon that was attributed by the authors to the gradual decay of initially-high SERCA activity^11^. In a comparable study using isolated rat RV trabeculae, the change in the force-frequency profile with time was revealed to be primarily associated with the time-dependent decrease of the sarcoplasmic reticular Ca^2+^ load^12^. To account for this unavoidable phenomenon, we have utilised a fully-balanced experimental design for the order of presentation of stimulus frequencies. We chose eight different frequencies spanning the spectrum of physiological heart rates of the rat (0.1 Hz to 10 Hz). This ‘Latin Square’ design ensures that, across all eight muscles, each of the eight stimulus frequencies both precedes and follows every other frequency precisely once. The virtue of this design resides in rendering the effect of time orthogonal to that of stimulus frequency. Thus, each of the eight stimulation sequences contains all eight stimulus frequencies. Hence, if muscle performance decreases with time, it will be revealed by the independent effect of order. This design has allowed us to investigate whether there is ‘run-down’ and, if so, whether it is ventricle-independent.

The second feature of our experimental protocols involves the inclusion of an intervention that allows assessment of ‘rested-state contractions’ in order to provide an independent test of the effect of time. In a rested-state contraction, the first twitch developed following a defined period of quiescence from a pause of stimulation is much greater in magnitude (potentiated), reflecting primarily a greater Ca^2+^ loading of the sarcoplasmic reticulum. We hypothesise that the post-rest potentiated twitch will decrease with stimulation order, consistent with the reported gradual diminution of sarcoplasmic reticular Ca^2+^ content^12^, and will also be ventricle-independent.

## Methods

### Ethical approval

Experiments were conducted in accordance with protocol R1341 approved by the Animal Ethics Committee of The University of Auckland. The protocol involved anaesthetising adult male Wistar rats (8-10 weeks old, 250–350 g) with isoflurane (1000 IU/kg), followed by cervical dislocation and cardiectomy.

### Muscle preparation

The excised heart was immediately Langendorff-perfused with modified Tyrode solution (in mmol L^−1^: 130 NaCl, 6 KCl, 1 MgCl_2_, 0.5 NaH_2_PO_4_, 0.3 CaCl_2_, 10 HEPES, 10 glucose and 20 2,3-butanedione monoxime). The pH of the Tyrode solution was adjusted to 7.4 at room temperature (22 °C) by using Tris and the solution continuously bubbled with 100% oxygen.

The heart was completely submerged in Tyrode solution. Under a microscope, trabeculae were dissected from the endocardial surfaces of both the left and right ventricles. Each geometrically-suitable trabecula was then transferred to a calorimeter. It was mounted between two platinum hooks, one connected to a force transducer at the downstream end with the other attached to a length-control motor at the upstream end. The trabecula was superfused with oxygenated Tyrode solution with a higher concentration of CaCl_2_ (1.5 mmol L^−1^) and in the absence of 2,3-butanedione monoxime. The superfusate was Tris-adjusted to pH 7.4 for body temperature (37 °C). The rate of flow of Tyrode superfusate was electronically maintained at 0.5 μL s^−1^. This flow rate provides adequate oxygenation to the muscle^13^ while maximising the thermal signal-to-noise ratio^14^.

### Experimental protocols

The trabecula was electrically stimulated via a pair of platinum electrodes located proximate to the calorimeter measurement chamber. It was stimulated to contract at 3 Hz for at least 1 hr to achieve mechanical and thermal steady states before it was gradually and incrementally stretched to optimal length to achieve maximal developed isometric force (*L*_*o*_). The entire calorimeter system was then enclosed within an insulated hood to diminish external optical and thermal disturbances. Body temperature was achieved by using the temperature controllers on the top and at the bottom of the calorimeter measurement unit, and from that installed in the optical table upon which the entire calorimeter system is mounted^15,16^.

Experiments commenced when a stable thermal environment had been attained. Each trabecula was stimulated to contract isometrically at eight different stimulus frequencies (0.1, 0.5, 1, 2, 3, 5, 7 and 10 Hz). The order of presentation of stimulus frequencies for an individual muscle was dictated by a fully-balanced 8 × 8 Latin Square. As shown in Table 1, each muscle was assigned a unique order of stimulus frequency that achieved full balance across all eight muscles. For example, muscle #1 experienced 5 Hz following 3 Hz, whereas muscle #5 experienced the converse order.

**Table 1:** The 8 × 8 Latin Square design for two groups of 8 trabeculae from each ventricle.

Stimulation Order	Muscle 1 (Hz)	Muscle 2 (Hz)	Muscle 3 (Hz)	Muscle 4 (Hz)	Muscle 5 (Hz)	Muscle 6 (Hz)	Muscle 7 (Hz)	Muscle 8 (Hz)
**1**	3	5	0.5	1	10	0.1	7	2
**2**	5	0.5	1	10	0.1	7	2	3
**3**	2	3	5	0.5	1	10	0.1	7
**4**	0.5	1	10	0.1	7	2	3	5
**5**	7	2	3	5	0.5	1	10	0.1
**6**	1	10	0.1	7	2	3	5	0.5
**7**	0.1	7	2	3	5	0.5	1	10
**8**	10	0.1	7	2	3	5	0.5	1

Each of the 8 muscles was assigned a unique order of presentation of stimulus frequencies. Note that in each row of ‘stimulation order’ and in each column of ‘muscle’, a specific frequency appears only once.

The developed isometric force and the rate of suprabasal heat output of each contracting trabecula were simultaneously recorded. Between consecutive stimulus frequencies, the trabecula was allowed to rest for a 2-min period by halting stimulation. Results were obtained from 16 trabeculae from each ventricle, arranged as duplicated 8 × 8 Latin Squares, hence requiring a total of 32 experiments. Trabeculae with developed steady-state stress of at least 25 kPa at the lowest frequency (0.1 Hz) were deemed acceptable for inclusion in the study.

Upon completion of an experiment, the trabecula was removed from the calorimeter. In its absence, the heat artefact resulting from electrical stimulation at various frequencies was quantified. The rate of muscle heat production at each frequency was then corrected retrospectively by subtraction of the electrical stimulus heat artefact.

### Muscle geometry

Muscle length and diameter were measured at *L*_*o*_ in the calorimeter from the top view using a microscope graticule. The geometry of each trabecula was assumed to approximate that of a cylinder of circular cross-section. The average dimension of the LV trabeculae (*n* = 16) did not differ significantly from that of the RV (*n* = 16) in either radii (116.8 μm ± 7.8 μm versus 102.3 μm ± 8.2 μm) or lengths (3.2 mm ± 0.2 mm versus 2.8 mm ± 0.2 mm).

### Definitions of measured variables

Force was converted to stress (kPa) by normalising to the estimated muscle cross-sectional area. Diastolic stress (kPa) was defined as the difference between the baseline of developed stress during a twitch and that recorded during the 2-min period of rest. Active stress (kPa) was defined as the peak stress developed above the diastolic level. Heat per twitch (kJ m^−3^) was calculated by dividing the steady-state rate of heat production (kW m^−3^) by the stimulus frequency and normalising by the estimated muscle volume. Stress-time integral (STI, kPa s) was defined as the area under the twitch stress profile. Maximal rates of rise (+d*S*/d*t*) and fall (−d*S*/d*t*) of steady-state stress were calculated from the ascending and descending limbs of the twitch.

### Statistical analysis

Measurements of force, length, and rate of heat output were acquired using LabVIEW software (National Instruments, Austin, USA) and analysed offline using a custom-written MATLAB (MathWorks, Natick, MA, USA) program. Measured variables were subjected to Analysis of Variance (ANOVA) using the SAS software package (SAS Institute Inc., Cary, NC, USA). The significance of differences between the two groups (LV and RV) was tested by examination of the Main Effects of ‘Latin Square’, ‘Ventricle’, ‘Order’, and ‘Stimulus Frequency’, as well as for the interaction effect between Ventricle and Frequency. The statistical significance of a difference was declared when *P* < *0*.*05*. Values are expressed as means ± standard errors.

## Results

### Effect of stimulus frequency

**Figure 1:**
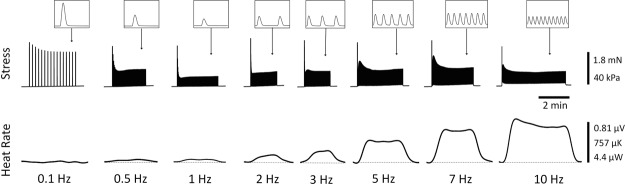
Typical experimental records of simultaneous measurements of stress and heat rate. Upper panel: twitch stress, where the insets depict individual steady-state twitches. Lower panel: rate of heat production, where the dotted line segments were drawn by connecting the silent periods between twitch trains to signify a heat-rate baseline. Peak stress developed during the first twitch at each frequency defines the rested-state contraction following a 2 min pause of stimulation. Data were obtained from a representative LV trabecula (length, 2.5 mm; diameter, 238 μm). For this muscle, the order of presentation of frequencies was 2, 3, 7, 5, 0.1, 0.5, 10, 1 Hz.

Figure 1 shows original records of the effects of stimulus frequency on the stress production and the rate of heat output of isolated trabeculae. The records show isometric contractions upon stimulation until steady state where stimulation was halted. The first twitch stress at each frequency upon stimulation was of the highest magnitude, representing the post-rest twitch potentiation. The steady-state stress was highest at the lowest frequency, but the rate of heat output was highest at the highest frequency. Note that at the two lowest frequencies, the rates of heat output were below the thermal resolution of the calorimeter, so were not analysed further for any of the 32 trabeculae examined. Note also that, at high frequencies, twitch relaxation was incomplete, resulting in increased diastolic stress. The frequency-dependent increase of diastolic stress was not different between RV and LV trabeculae (data not shown).

**Figure 2:**
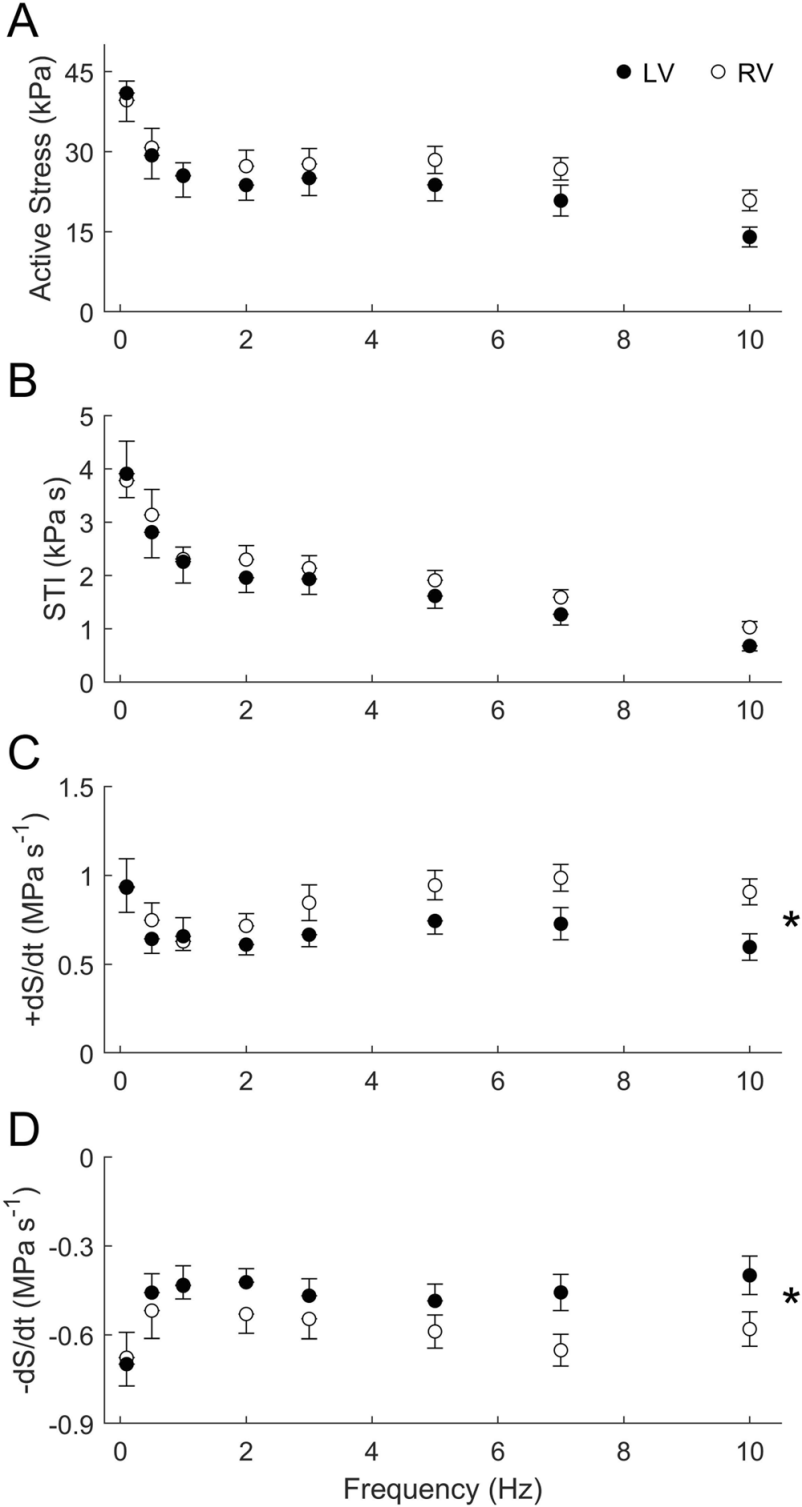
Twitch characteristics of RV and LV trabeculae at steady-state under isometric contractions. Averaged active stress (**A**), stress-time integral, STI (**B**), maximal rates of twitch stress development (+dS/dt, **C**) and relaxation (−d*S*/d*t*, **D**) are presented as functions of stimulus frequency. The star symbol denotes a significant interaction between ventricle and frequency.

Averaged data of twitch characteristics during steady-state isometric contractions are shown in Fig. 2. The magnitude of active twitch stress (Fig. 2A) and the area under the twitch (stress-time integral, STI, Fig. 2B) across all muscles both decreased with stimulus frequency, but with no difference between the ventricles. Trabeculae from the RV showed significantly greater rates of rise and fall of twitch stress (±d*S*/d*t*) than those of the LV. These ventricle-dependent effects were more pronounced at high frequencies (7 Hz and 10 Hz), as revealed by the significant interaction terms in the ANOVA and indicated by the star symbol (Fig. 2C,D).

**Figure 3:**
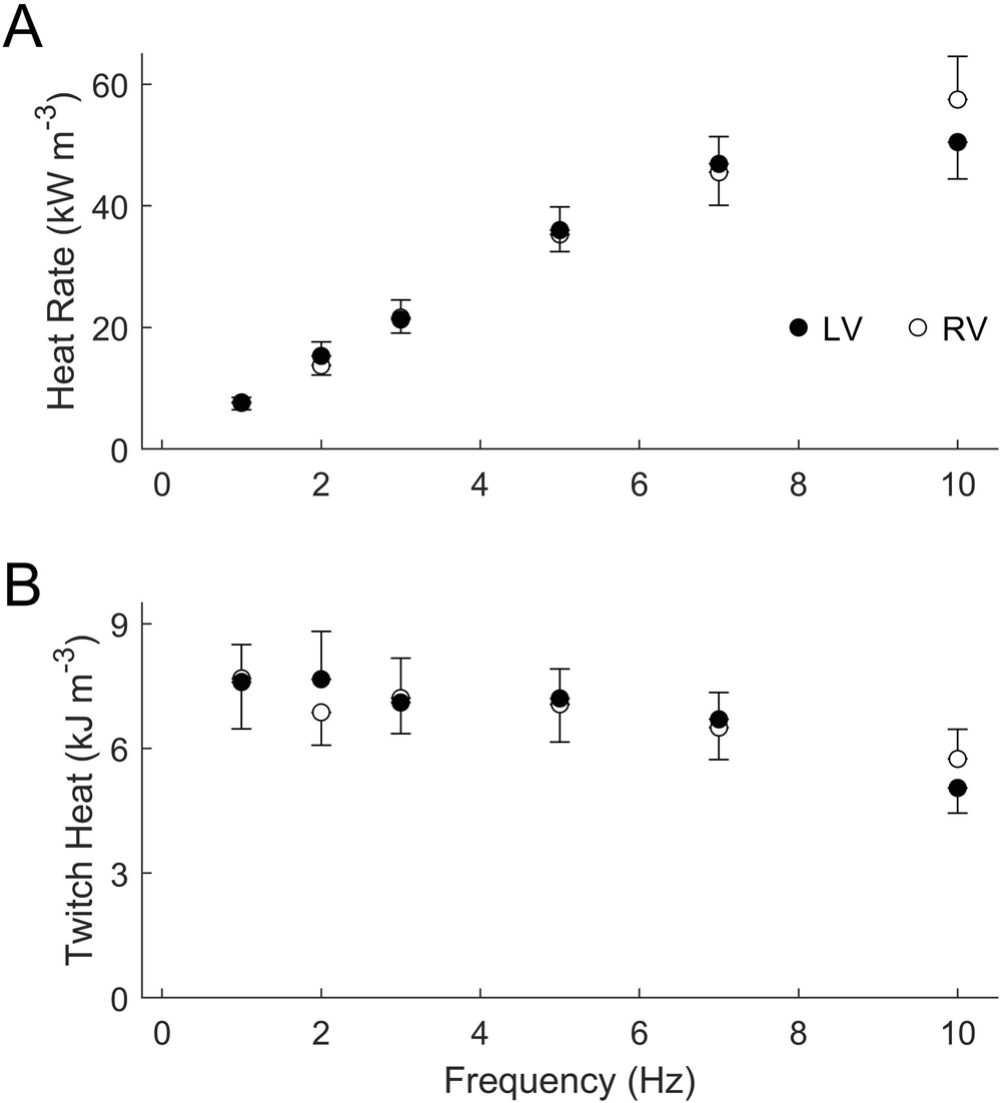
Heat outputs of RV and LV trabeculae at steady-state under isometric contractions. Averaged rate of heat output (**A**) and heat per twitch (heat rate divided by stimulus frequency; **B**) are plotted as functions of stimulus frequency.

**Figure 4:**
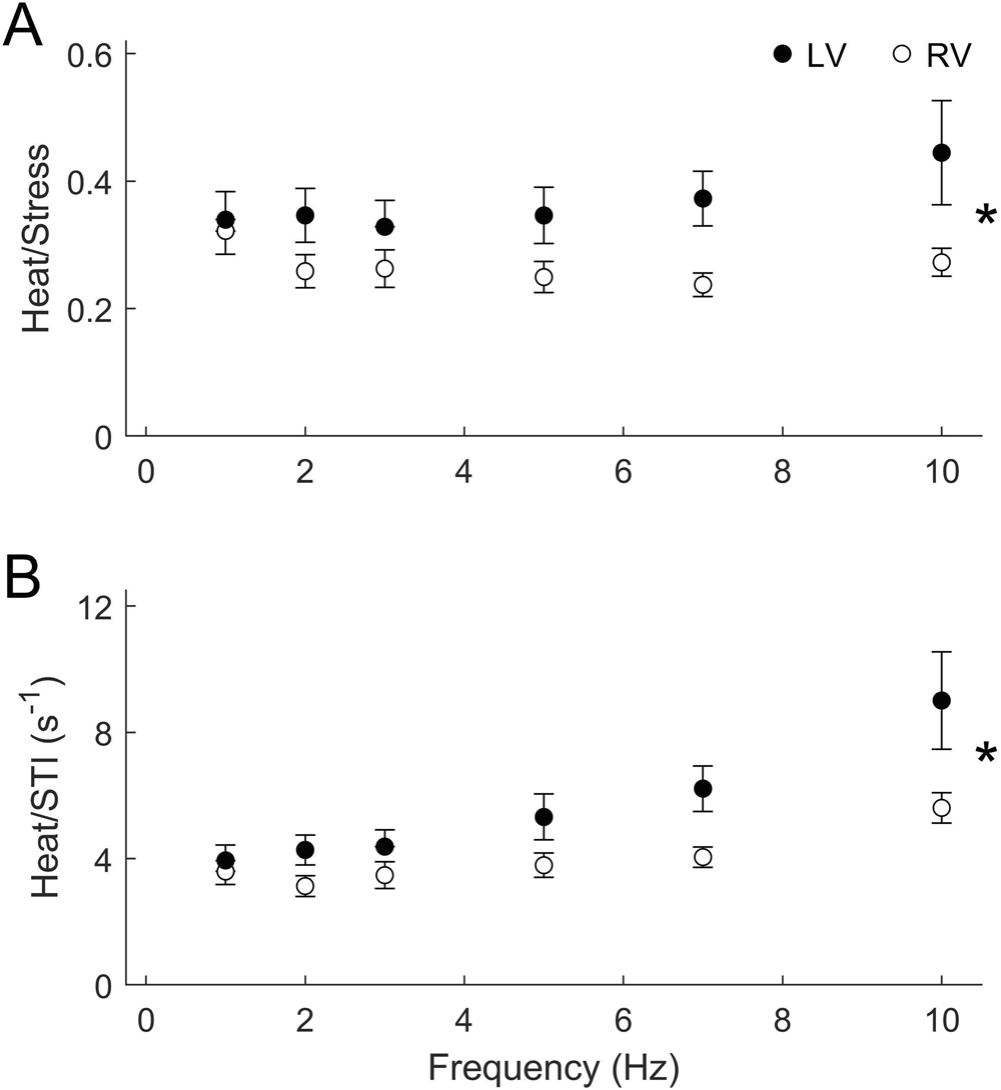
Mechanoenergetic indices of RV and LV trabeculae at steady-state under isometric contractions. The ratio of heat to active stress (**A**), and that of heat to STI (**B**), of each trabecula in either ventricle group were averaged and the averages presented as functions of stimulus frequency. The star symbol denotes a significant interaction between ventricle and frequency.

There were no statistically significant differences in either steady-state heat rate (Fig. 3A) or heat per twitch (Fig. 3B) between muscles from the two ventricles. However, when heat was normalised either to active stress (Fig. 4A), or to stress-time integral (Fig. 4B), trabeculae from the LV showed significantly greater normalised heat than those from the RV over the range of frequencies examined. The higher normalised heat of the LV trabeculae is due to their lower values of active stress or stress-time integral, despite the absence of difference between ventricle when tested across the full range of stimulus frequencies (Fig. 2A,B).

### Effect of time

**Figure 5:**
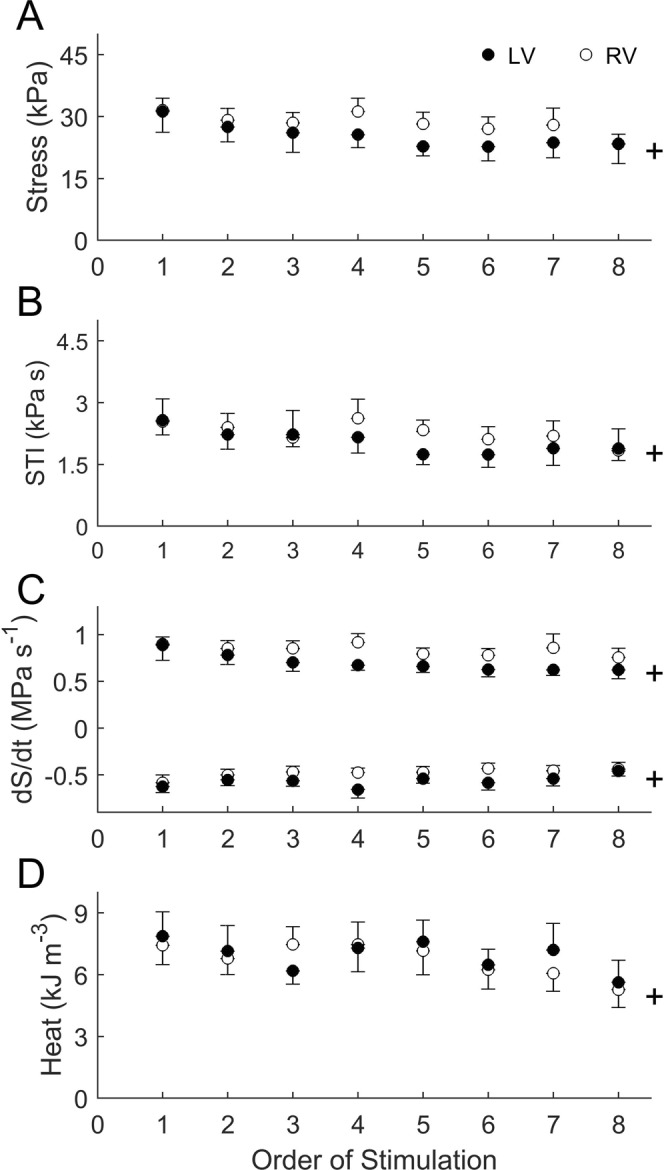
Effect of time on muscle mechanoenergetic performance. For each dependent variable: active stress (**A**), stress-time integral (STI) (**B**), rates of rise and fall of stress (**C**), and heat (**D**), all values were averaged across frequencies for each order of stimulation. The ‘+’ symbol denotes a significant effect of time.

Figure 5 shows the average values of each variable as functions of orders of stimulation (i.e., as functions of time). There were significant time effects in the steady-state stress, stress-time integral, twitch kinetics, and heat output. However, the profiles of decline of these variables were ventricle-independent.

**Figure 6:**
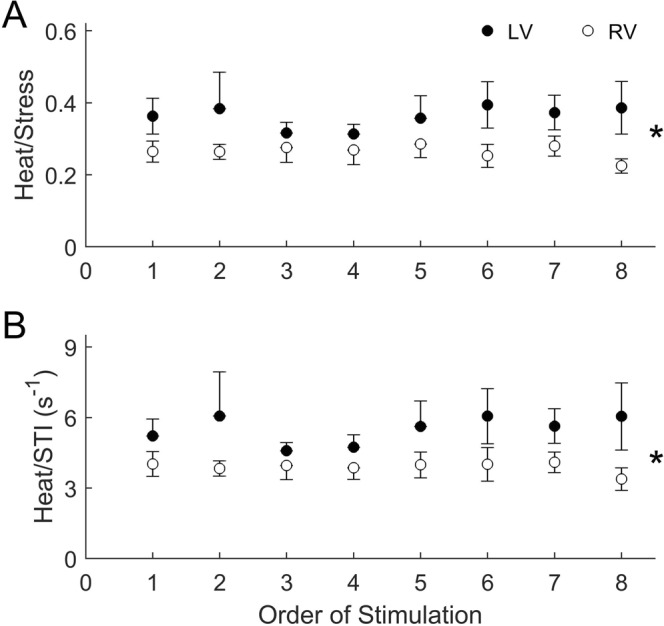
Steady-state heat per stress and per stress-time integral as functions of time. For each dependent variable, heat per stress (**A**) and heat per stress-time integral (**B**), there were no effects of time but there was a difference between the ventricles (denoted by the ‘*’ symbol).

As shown in Fig. 6, there were no significant time effect on either the ratio of heat to stress or heat to stress-time integral in either ventricle. This was because stress, stress-time integral, and heat concomitantly declined with time (Fig. 5). Although these ratios were independent of time, they were dependent on ventricle, with LV trabeculae having higher values than RV trabeculae as assessed across all stimulus frequencies.

**Figure 7:**
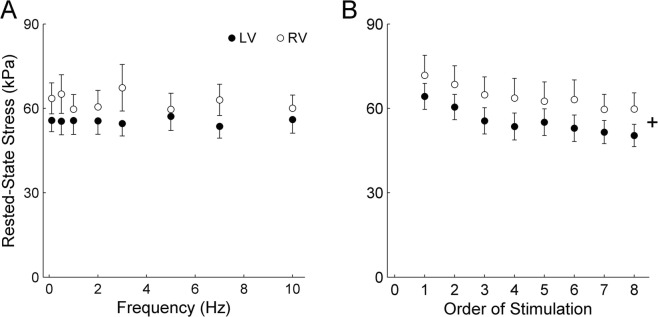
Rested-state potentiated stress. The average values of rested-state stress (**A**) are presented as functions of stimulus frequency. Rested-state stress data, averaged across all eight frequencies, are plotted as a function of time (order), showing a progressive decline with time (as indicated by the ‘+’ symbol) independent of ventricle (**B**).

Figure 7 shows a time-dependent, but ventricle-independent, decline of rested-state stress throughout the duration of the experiments. Figure 7A also demonstrates the frequency-independent effect of rested-state potentiated stress production.

## Discussion

The present study is the first to compare the energy expenditure between left-ventricular (LV) and right-ventricular (RV) myocardial preparations contracting isometrically over a wide range of stimulus frequencies encompassing the physiological heart rate of the rat. This comparison has been made possible by our simultaneous measurements of active stress production and heat output (Fig. 1) of trabeculae at body temperature. We are thus able to assess the effect of ventricle and stimulus frequency on the isometric stress production (Fig. 2), heat output (Fig. 3), and their ratios (Fig. 4). In addition to the effects of ventricle and frequency, our Latin Square experimental design (Table 1) inherently allows an independent examination of the effect of time on muscle performance. Whereas this design revealed that active stress (Fig. 5) and heat output (Fig. 6), whether from steady-state or rested-state (Fig. 7) contractions, declined with time, this decline could be assessed independently of the effect of stimulus frequency.

The time-dependent decline of stress in isolated cardiac muscle preparations has been attributed to a decay of SERCA activity^11^ as well as a decrease of Ca^2+^ content in the SR^12^. It is well accepted that the SR Ca^2+^ content plays a critical role in the production of stress after long rest periods^17,18^. Upon the recommencement of stimulation after a period of rest, the first isometric twitch displays “post-rest potentiation” (Fig. 7). The development of such rested-state stress is dependent upon the SR Ca^2+^ load and the amount of Ca^2+^ released per twitch^17,18^. In the rat, rested-state stress at body temperature increases progressively with the duration of the rest intervals and reaches saturation after 0.5-1 min of rest^4,12,17^. We designed our experiments to allow trabeculae to rest for 2 min, thereby providing confidence that the maximal post-rested stress was achieved. Consistent with the time-dependent loss of SR Ca^2+^ load, our results show that the rested-state active stress also declined with time (Fig. 7B). The decline could also be associated with the time-dependent loss of Ca^2+^ sensitivity of the myofilaments^19^. We show that the extent of decline of isometric twitch stress with time is ventricle-independent, suggesting the time-dependent loss of SR activity and Ca^2+^ load, as well as myofilament Ca^2+^ sensitivity, are events occurring simultaneously in both RV and LV ventricular myocardia post-dissection, *in vitro*. Given that these events involve SERCA and crossbridge ATPases, we show that the bulk of heat output associated with these ATPase activities also declined with time. What is noteworthy is that when heat is normalised to stress or to stress-time integral, there is no change of normalised heat with time (Fig. 6). This finding reveals that the economy of contraction is independent of time.

Economy of contraction denotes the amount of energy expenditure, typically measured as heat output, for a given stress production during isometric contractions. This index has been used to assess the energy cost of isometric force production with aging^20^, at different temperatures^21^ and Ca^2+^ concentrations^19^, as well as in cardiac hypertrophy^22,23^. We found that although economy is independent of time, it is dependent on ventricle (Fig. 6). Our result shows that RV trabeculae are more economical, i.e., the heat output of RV trabeculae for a given unit of stress is lower, than that of the LV trabeculae. This result holds also when heat is normalised by stress-time integral. The rationale of normalising heat to stress-time integral is that active heat output is correlated not only with active stress^9,19,24,25^, but has also with stress-time integral^26–29^.

Whereas economy is used for assessing cardiac energetics in intact muscle preparations, tension cost is the term commonly employed for skinned myocardium. Tension cost refers to the ratio of rate of ATPase activity to tension or force. These energetic indices, although different in terminology, are conceptually the same. That is, decreased tension cost implies that economy is increased. Using either intact or skinned cardiac preparations, it has been demonstrated that economy increases with age^20,30^ as well as in various diseases. Such diseases include pressure-overloaded ventricular hypertrophy^22,31^, hypothyroid-induced ventricular atrophy^23,32–34^, and diabetes^35^. At the whole-heart level, the isovolumically-beating hearts of spontaneously hypertensive rats show a lower ratio of left-ventricular developed pressure to oxygen consumption than their control Wistar-Kyoto rats^36^, suggesting a greater economy of contraction. These results, showing improved economy, are obtained where a concurrent slowing of contraction kinetics is evident. Slowed kinetics has been ascertained from a reduction of maximal dS/dt^22,23^, paralleled by a shift in the myosin heavy chain isoform (MHC) from predominantly α (fast) to predominantly β (slow)^23,30–35^. These observations have led to the notion that slowing of contractile kinetics is an adaptive response that increases the economy of contraction^22,23,31,33,34,37,38^.

Our results allow us to reject the notion discussed in the preceding paragraph. We find that rat LV trabeculae, compared with RV trabeculae, have slower kinetics, as manifested by their lower rates of rise and fall of twitch stress (Fig. 2). Their slower twitch kinetics is consistent with a greater proportion of β-MHC in the LV myocardium compared with that of the RV myocardium^39–41^, as well as with lower shortening velocity in the LV muscles compared with that of RV muscles^5,9,41^. Despite their slower kinetics, we observed no greater economy of contraction. Rather, their economy was lower than that of the RV trabeculae (Fig. 4). We speculate that the discrepancy between our results and those that led to the aforementioned notion is because our results are obtained from healthy tissues where the β-MHC isoform abundance is naturally greater in the LV myocardium than in the RV. That is, the LV is inherently slower than the RV in the healthy heart. Contrariwise, in aging and in disease, although there is a progressive shift from predominantly α-MHC to predominantly β-MHC isoform, there are likely to be other concurrent mechanisms or parallel compensatory adaptations that lead to an improvement in contractile economy. Our results, obtained from healthy tissues, reveal that slow contraction kinetics alone are not a causative mechanism for improvement of economy. It is noteworthy that the aforementioned notion is predicated on correlational, rather than causative, evidence. It is thus that the often-observed slowing of contractile kinetics in aging and in disease may have been inappropriately assumed to be a causative mechanism for the improvement of economy of contraction. Our results provide direct evidence that the parallel between changes in the kinetics of contraction and the economy of contraction is not a causal link between mechanical performance and energetic adaptation, at least in healthy myocardium.

We conclude that, over a physiological range of stimulus frequencies, despite slower contractile kinetics in the left-ventricular myocardium, the energy expenditure during isometric contractions is ventricle-independent. Economy of contraction, on the other hand, is lower in the left-ventricular muscles, as a function of stimulus frequency as well as a function of experimental duration.
